# Surgical management and outcome of rectal carcinoids in a university hospital

**DOI:** 10.1186/s12957-015-0463-3

**Published:** 2015-02-07

**Authors:** Rockson Wei, Oswens SH Lo, Wai Lun Law

**Affiliations:** Division of Colorectal Surgery, Department of Surgery, The University of Hong Kong, Queen Mary Hospital, Pok Fu Lam, Hong Kong

## Abstract

**Background:**

Rectal carcinoids are an uncommon entity comprising only 1%–2% of all rectal tumors. Rectal carcinoids are frequently diagnosed during colonoscopy, but management after polypectomy is still controversial. The aims of this study were to review the surgical procedures for rectal carcinoids and to compare the outcomes of patients after different treatment modalities in a university hospital in Hong Kong.

**Methods:**

All rectal carcinoids diagnosed between January 2003 and September 2012 were reviewed retrospectively, including clinicopathological characteristics, their management, and surgical outcomes.

**Results:**

There were 54 patients with a median age of 60 years, and 32 were males (59.3%). All patients underwent colonoscopy, and the most had rectal bleeding (53.7%). Two patients were diagnosed incidentally in the surgical specimens of rectal tissues. Eighteen patients were diagnosed to have rectal carcinoids after snaring polypectomy, and no further intervention was required. Twenty-five patients had local resection either by means of transanal resection or transanal endoscopic operation. Radical resection was performed in seven patients in which one had T3N1 disease and the others did not have any lymph node metastasis.

In the median follow-up of 30 months (10–108 months), there was no recurrence in the “incidental” or post-polypectomy group. However, two patients with transanal resection and two patients with radical resection developed hepatic metastases after 13–24 months post-treatment. The 5-year overall survival was 100% in patients having snaring polypectomy only, 83% for those with local resection, and 63% in patients who underwent radical surgery (*p* = 0.04).

**Conclusions:**

Our data suggested that that local resection was an effective treatment for small rectal carcinoids and generally brought about good oncological and surgical outcomes. For larger tumors, radical resection seemed to provide acceptable oncological outcomes. Regular surveillance with colonoscopy and endorectal ultrasound is highly recommended for high-risk patients for long-term management. By sharing our experience, we hope to provide more evidence on the management on rectal carcinoids which, together with evidence from further studies, may guide us in the long-term management of these patients in the future.

## Background

Carcinoid tumors, a type of neuroendocrine tumors, comprise of a heterogeneous group of neoplasms arising from enterochromaffin cells. These relatively uncommon tumors have variable histological patterns and biologic behaviors [1,2]. Rectum is the third most common site of occurrence for carcinoids, with up to 13.7% of all carcinoids found at that site. On the other hand, carcinoids only comprise of 1%–2% of all rectal tumors [3,4]. Both the overall incidence of carcinoid tumors as well as the proportion of rectal carcinoids in all rectal tumors has been increasing drastically in recent years [3,4]. The ageing population, increased rate of screening with colonoscopy, and the increased awareness of the clinicians probably account for this change in incidence [3]. Concerning treatment, studies of different populations showed that sizes of rectal carcinoid lesions are usually small [2-4]. While larger lesions require major rectal resection, most can be treated with local excision either using endoscopic means or via transanal route with good surgical outcomes [5,6]. However, there is still no consensus on the treatment strategy and surveillance of rectal cancer in general yet [5,7,8].

The aims of this study were to review the surgical procedures for rectal carcinoids and to compare the outcomes of patients after different treatment modalities in a university hospital in Hong Kong.

## Methods

### Patient inclusion

All patients who had treatment of rectal carcinoids at Queen Mary Hospital between January 2003 and September 2012 were included in this study. All tumors were located within 15 cm from anal verge. Data on patients’ demographics, clinical presentations, treatment modalities, recurrence, and survival were retrospectively reviewed and analyzed. The tumor size was defined by the largest diameter of the tumor. Other histopathological characteristics, such as resection margins, mitotic rate, and immunostaining features, were searched for and analyzed if available.

### Treatment methods

Endoscopic treatment such as snaring polypectomy or endoscopic mucosal resection resulting in adequately clear margins was considered as curative treatment for small rectal carcinoids (<10 mm). For larger rectal carcinoids (>10 mm) or those with positive margin after polypectomy, repeated resection was performed. Local excisional procedures, either transanal resection or transanal endoscopic operation (TEO), would be performed. Radical rectal resection (such as low anterior resection) was performed in patients with potentially aggressive carcinoids or concomitant rectal adenocarcinoma.

All patients were followed up in outpatient clinic with digital examinations performed at 2 to 4 weeks after operation, and then at every 2–3-month intervals in the first and second year. Follow-up appointments would then be spaced out to yearly afterwards. We offer lifelong follow-up assessment. Surveillance colonoscopy would be arranged to look for any local recurrence or metachronous colorectal malignancy in a 3-yearly interval. CT scan was used only in 3- to 5-year intervals to look for distant metastasis in patients who presented with poor prognostic features.

### Statistical methods

Data were analyzed with PASW version 18. Continuous variables were analyzed with *t*-test, whereas categorical variables were compared with chi-square test. Kaplan Meier survival curve was used for survival analysis, and comparison was made with log-rank test. *p* values of less than 0.05 were considered significant.

## Results

During the study period, there were 54 patients diagnosed to have rectal carcinoids and all were of Chinese ethnicity. Thirty-two patients were males (59.3%), and the median age was 61 among them all (range: 29 to 88 years). The most common presenting symptom was rectal bleeding (53.7%). Only one patient had symptoms of carcinoid syndrome with spurious diarrhea and flushing. In large carcinoid tumors, (>10 mm), statistically significant association was shown with symptoms of anal pain or discomfort (*p* = 0.024) but not with bleeding (*p* = 0.349) (Table 1).

**Table 1:** **Data on patients’ characteristics**

	**Overall**	**Size <10 mm**	**Size <10 mm**	***p*** **values**
Age (median)	60	57	65	0.716
Male:female	32:22	26:16	6:6	0.382
Presenting symptoms				
Bleeding	28 (51.9%)	20 (37.0%)	8 (14.8%)	0.349
Tenesmus	6 (11.1%)	4 (7.4%)	2 (3.7%)	0.547
Anal/rectal mass	7 (13.0%)	3 (5.6%)	4 (7.4%)	0.024
Anal pain/discomfort	7 (13%)	3 (5.6%)	4 (7.4%)	0.024
Carcinoid syndrome	1 (1.9%)	0 (0%)	1 (1.9%)	0.069
Treatment modalities				
Snaring polypectomy	18 (33.3%)	0 (0%)	18 (33.3%)	
Transanal resection	10 (18.5%)	4 (7.4%)	6 (11.1%)	
TEO	15 (27.5%)	3 (5.6%)	12 (22.2%)	
Radical resection	7 (13.0%)	3 (5.6%)	4 (7.4%)	
No treatment	4 (7.4)	2 (3.7%)	2 (3.7%)	

In most patients, the diagnosis of carcinoid was made by endoscopic examination and biopsy except for two patients who were diagnosed to have rectal carcinoids after surgical procedures for seemingly unrelated diseases; one after hemorrhoidectomy and the other after the transrectal prostate biopsy, with the specimens showing the presence of rectal carcinoid.

Most gastrointestinal carcinoids can be recognized during routine microscopic examination, but immunohistochemical staining is a useful aid. Chromogranin A, a glycoprotein stored in secretory granules of neuroendocrine cells, was positive in 75.6% of the patients. Synaptophysin, a neuron-specific enolase test, was found positive in 97.9% of the patients.

The median size of the rectal carcinoids was 5 mm, and it ranged from 1 to 60 mm. Seventeen patients with small tumors were treated by snaring polypectomy alone, and one patient had endoscopic mucosal resection. The median size of the tumor in this group of patients (group 1) was 4.5 mm (range: 1 to 8 mm). The indications for further intervention after snaring polypectomy included incomplete endoscopic removal or positive resection margins (Table 2), and the 25 patients belonging to this group (group 2) underwent either transanal resection (18.5%) or TEO (27.8%). The median tumor size in this group of patients was larger than that in the previous group, being 6 mm (range: 1.5 to 25 mm). The third group (group 3) consisting of seven patients (13.0%) who had radical resection for tumors of larger size, concomitant rectal adenocarcinoma, or margin involvement (Table 2). Of these seven patients, one had T3N1 disease and the other had T2 or less without lymph node metastasis. Two other patients had metastatic disease on presentation and therefore did not undergo any surgical treatment.

**Table 2:** **Indications for further intervention after snaring polypectomy**

	**Inadequate margins**	**Large size**	**Abnormal imaging**	**Submuscosal lesion**	**Concomitant tumor**	**Total**
Snaring polypectomy only	N/A	N/A	N/A	N/A	N/A	18 (33.3%)
Transanal resection	4 (7.4%)	4 (7.4%)	1 (1.9%)	1 (1.9%)	0 (0%)	10 (18.5%)
TEO	8 (14.8%)	5 (9.3%)	0 (0%)	2 (3.7%)	0 (0%)	15 (27.8%)
Radical resection	0 (0%)	3 (5.6%)	0 (0%)	0 (0%)	4 (7.4%)	7 (13.0%)

*N/A* not applicable.

During the median follow-up of 30 months (10 to 108 months), no local or systemic disease recurrence was detected in the polypectomy alone group (group 1). In the local resection group (group 2), three patients had disease recurrence after transanal resection. None of the patients who underwent TEO had disease recurrence. One patient had local recurrence after 24 months and was disease free for 35 months after another transanal resection. The other two patients developed and subsequently died of systemic recurrence, one at 5 months and the other at 13 months after initial operation. In the radical resection group (group 3), one patient developed local recurrence and two patients had liver metastases within 1 year postoperatively. The other four patients underwent low anterior resection for synchronous rectal adenocarcinoma (7.0%), and all were disease free with median follow-up of 2 years.

**Figure 1:**
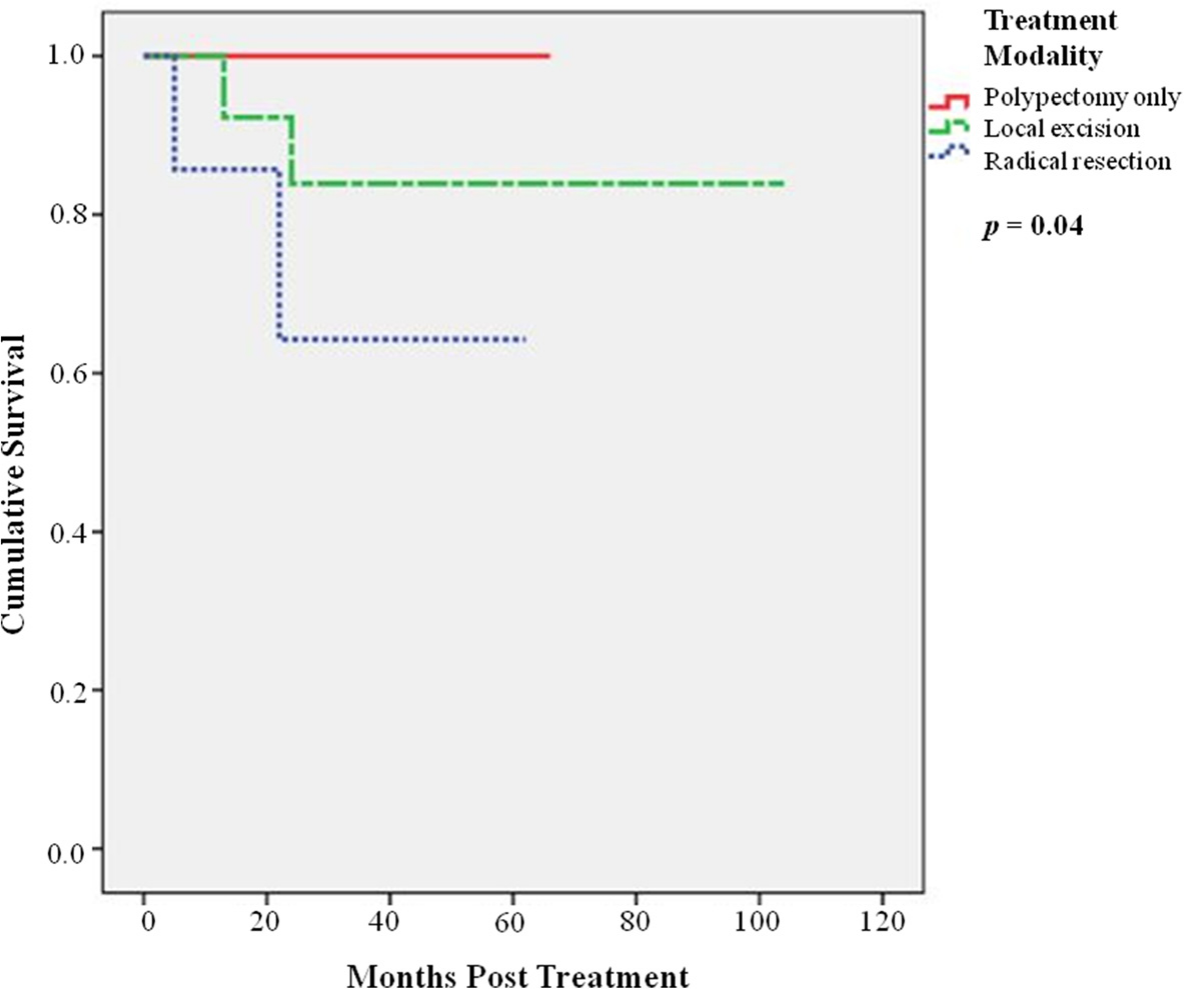
**Patients’ overall survival following snaring polypectomy, with local resection, and with radical resection (** ***p*** **= 0.04).**

**Figure 2:**
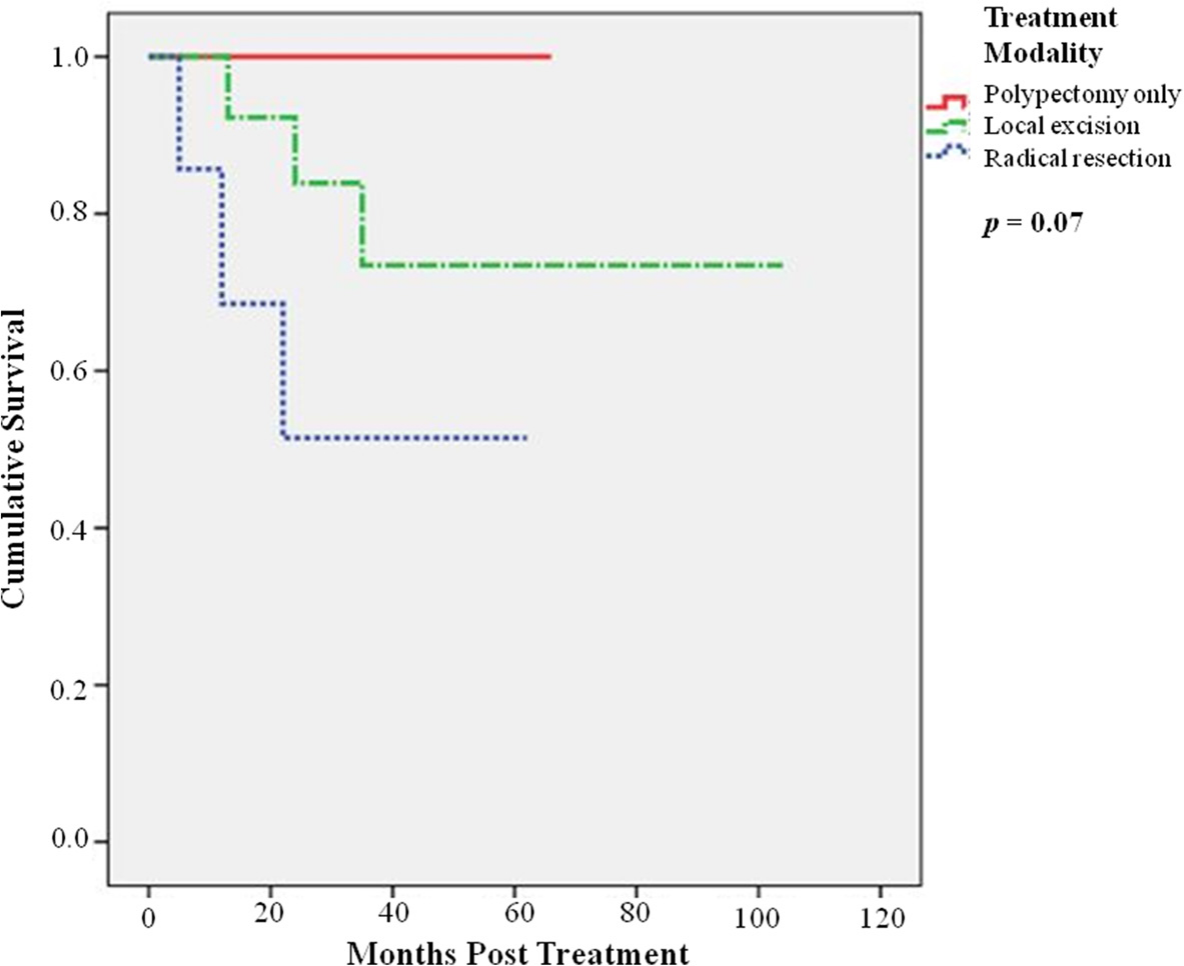
**Patients’ disease-free survival following snaring polypectomy, with local resection, and with radical resection (** ***p*** **= 0.07).**

**Figure 3:**
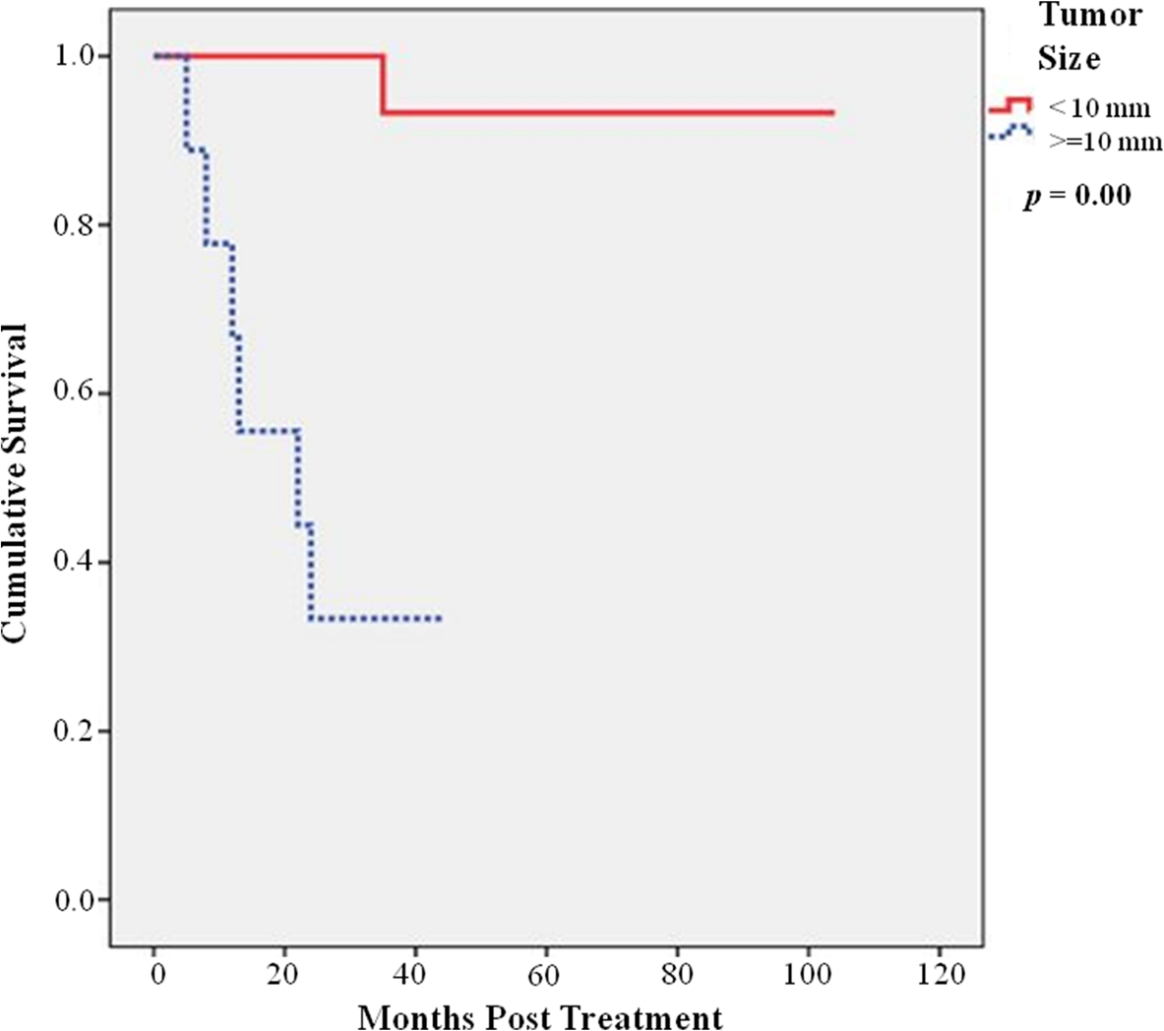
**Patients with tumor size of more than 10 mm showed significantly worse 5-year disease-free survival.**

Subsequently, in overall six patients died of disease and their demographics were summarized in Table 3. The most common site of distant metastases was liver (100%), followed by bone (33%), lung (17%), and pancreas (17%). The overall 5-year disease-free survival was 87.0%. Concerning different treatment methods, the 5-year overall survival was 100% in patients following snaring polypectomy, 83% for those with local resection, and 63% for those with radical resection (*p* = 0.04) (Figure 1), whereas the 5-year disease-free survival was 100% in patients following snaring polypectomy, 73% for those with local resection, and 51% for those with radical resection (*p* = 0.07) (Figure 2). Considering association of tumor size and prognosis, patients with tumor size of more than 10 mm showed significantly worse 5-year disease-free survival than those with less than 10 mm (37% vs 97.1%) (*p* = 0.001) (Figure 3).

**Table 3:** **Data on patients’ demographics who developed distant metastases**

**Age**	**Sex**	**Size (mm)**	**Treatment**	**Site of metastasis**	**Survival (months)**
48	M	34	LAR	Liver	22
53	M	25	Not treated	Liver	8
64	M	60	Not treated	Liver, bone	1
65	F	15	Transanal excision, chemotherapy after metastasis	Liver, bone	24
74	M	25	Transanal excision	Liver, pancreas	13
77	F	15	LAR	Liver	5

## Discussion

Rectal carcinoids are usually small at the initial presentation, and most studies reported a median size of 6 mm, compared with a median size of 1–3 cm in colonic carcinoids [5,9]. Similarly, the median size of the rectal carcinoids in this study was 5 mm, and it ranged from 1 to 60 mm. Patients with rectal carcinoids usually present with local symptoms. Many were diagnosed incidentally during endoscopy (31.5%). Carcinoid syndrome is associated with excessive secretion of serotonin, tachykinins, prostaglandins, catecholamines, and histamine. 5-Hydroxyindoleacetic acid (5-HIAA), their metabolite, was excreted in the urine, allowing it to be used as a diagnostic and postoperative surveillance marker. However, as hindgut carcinoid rarely secretes serotonin, carcinoid syndrome is uncommon and urinary 5-HIAA is seldom elevated [10]. This explains why in our study, only one patient had carcinoid syndrome and elevated urinary 5-HIAA (1/54). Immunohistochemical staining as an aid for the diagnosis of rectal carcinoid had also been explored in our study. Chromogranin A had a much lower sensitivity of 75.6% in our study, compared with 97.9% for synaptophysin. Eriksson et al. suggested using plasma chromogranin A for monitoring of disease progression in midgut neuroendocrine tumors (NETs) especially in patients with metastatic disease, but not in hindgut NETs, since there was limited evidence to suggest similar applicability [11].

Snaring polypectomy and endoscopic mucosal resection is an adequate treatment for most rectal carcinoids with small size [5,6]. In our study, one third of patients were diagnosed incidentally after endoscopic polypectomy, and no further intervention was needed. None subsequently developed local recurrence during follow-up. For larger tumors or those with margin involvement, local excision (either transanal excision or TEO) was performed. The latter approach could be employed for upper rectal lesions with better visualization and adequate margin with low morbidity [8]. However, both surgical approaches cannot remove potentially involved regional lymph nodes, which have been reported to have an incidence of up to 8.3% [7]. In our series, these patients had good outcome with a 5-year overall survival of 83% and 5-year disease-free survival of 72%, reflecting the adequacy of local treatment. From the limited number of patients who underwent local excision, we found that the use of TEO could achieve complete excision with clear margins. No patients had recurrence after the procedure. For large rectal carcinoids which required radical resection, the 5-year overall survival rate was 63% and disease-free survival was 50%. Radical resection for this group of patients was necessary due to larger tumor size and the possibility of loco-regional lymph node metastasis [7]. The study showed that the 5-year overall survival was 100% in patients following snaring polypectomy, 83% for those with local resection, and 63% for those with radical resection, which is comparable with the reported 5-year survival rate of about 88% for all tumor stages [5,12,13].

Size is an important and known prognostic factor for carcinoids, and those less than 10 mm are even considered benign [5,6,12,13]. Other important factors include the depth of invasion, lymphovascular invasion (LVI), and the number of mitotic figures [12,14-16]. Larger primary tumors in our study (>10 mm in size) were associated with a worse prognosis with 5-year survival of only 37%, and this was consistent with previous reports [2,5,12]. Our metastatic rate was 9.3%, consistent with other studies which ranged from 4% to 18% [12,17,18]. Mostly rectal carcinoids are localized with a low propensity of metastasis at the time of diagnosis, with the rate of metastasis ranging from 1.7% to 3.4% [6,19]. In the case of large tumors or perceived advanced disease, further imaging such as computed tomography, PET scans, or endorectal ultrasound is indicated [5,7].

Synchronous non-neuroendocrine tumors were reported in the literatures with incidences of 25% to 46% [20,21]. Interestingly, most synchronous tumors were observed in the gastrointestinal tract, whereas metachronous tumors were more often observed outside the gastrointestinal tract [13,20]. As the presence of a metachronous malignancy was associated with more aggressive behavior in NET, close surveillance program was suggested in these cases [22]. In our study, four patients had synchronous carcinoma of rectum, and none of them developed metachronous tumors or recurrence.

Few clear recommendations are available on post-treatment surveillance of NETs [5,7,23]. In our center, the patients were followed up 2–3 monthly in the first two postoperative years, followed by lifelong annual assessment afterwards. Though surveillance endoscopy in this context had not been widely documented in medical literature, regular colonoscopy would be considered every 3–5 years with an aim to rule out local recurrence and metachronous colorectal malignancy [7,23]. Although most local recurrences could be detected by digital examination and colonoscopy in our patients, some smaller submucosal recurrence could still be missed. Endorectal ultrasound in this case can play a role in identifying small submucosal recurrence, and it is recommended as a surveillance tool for monitoring local disease recurrence [7,23]. For those stage II/III diseases, surveillance with computed tomography would be essential to look for any distant metastases, particularly liver metastases, as we have been offering to our patients. As distant metastases may occur years after the initial treatment, long-term surveillance beyond 5 years had been suggested in patients with high-risk factors such as large size and advanced tumor staging with lymph node metastasis [23]. Chromogranin A is not commonly elevated in this group of patients, thus limiting their utilization [11].

## Conclusions

Our data suggested that that local resection was an effective treatment for small rectal carcinoids and generally brought about good oncological and surgical outcomes. For larger tumors, radical resection seemed to provide acceptable oncological outcomes. Regular surveillance with colonoscopy and endorectal ultrasound is highly recommended for high-risk patients for long-term management. By sharing our experience, we hope to provide more evidence on the management on rectal carcinoids which, together with evidence from further studies, may guide us in the long-term management of these patients in the future.
